# Synthesis of semicrystalline nanocapsular structures obtained by Thermally Induced Phase Separation in nanoconfinement

**DOI:** 10.1038/srep32727

**Published:** 2016-09-08

**Authors:** Enza Torino, Rosaria Aruta, Teresa Sibillano, Cinzia Giannini, Paolo A. Netti

**Affiliations:** 1 Center for Advanced Biomaterials for Health Care @CRIB - Istituto Italiano di Tecnologia (IIT), Largo Barsanti e Matteucci n. 53, 80125, Napoli, Italy; 2 University of Naples Federico II, Interdisciplinary Research Center of Biomaterials, CRIB P.le Tecchio 80, 80125, Naples, Italy; 3 University of Naples Federico II, Department of Chemical, Materials and Industrial Production Engineering, P.le Tecchio 80, 80125, Naples, Italy; 4 CNR - IC Istituto di Cristallografia, via Amendola 122/O, 70126 Bari-Italia

## Abstract

Phase separation of a polymer solution exhibits a peculiar behavior when induced in a nanoconfinement. The energetic constraints introduce additional interactions between the polymer segments that reduce the number of available configurations. In our work, this effect is exploited in a one-step strategy called nanoconfined-Thermally Induced Phase Separation (*nc-TIPS*) to promote the crystallization of polymer chains into nanocapsular structures of controlled size and shell thickness. This is accomplished by performing a quench step of a low-concentrated PLLA-dioxane-water solution included in emulsions of mean droplet size <500 nm acting as nanodomains. The control of nanoconfinement conditions enables not only the production of nanocapsules with a minimum mean particle diameter of 70 nm but also the tunability of shell thickness and its crystallinity degree. The specific properties of the developed nanocapsular architectures have important implications on release mechanism and loading capability of hydrophilic and lipophilic payload compounds.

The thermodynamics of macromolecules in a confined environment is extremely different from that of macroscopic systems; it has been object of numerous theoretical speculations, because of its importance in several fields including biology, medicine and nanotechnology^1^,^2^,^3^,^4^,^5^,^6^,^7^,^8^,^9^. Past investigations into phase separation of polymer solutions focused mainly on the crystallization kinetics and morphology of polymer crystals growing from *bulk* solutions. However, when confined in a reduced volume (nanoconfinement), the macromolecules of a polymer solution can exhibit a wide range of peculiar physical behaviors, leading to surface-driven phase changes, which include new kinds of transitions, not observed in the bulk^8^,^9^,^10^,^11^.

To date, mainly computational studies have been developed to unravel the interactions occurring at the interfaces of nanoconfined binary systems, such as solvent–solvent and polymer–solvent^12^,^13^,^14^,^15^,^16^,^17^,^18^,^19^. Comparatively, fewer experimental studies addressed the phase separation behavior of binary polymer solutions in a nanoconfined domain, and an even lower number of investigations have been addressed to ternary system polymer -solvent-antisolvent^9^,^11^,^17^,^18^,^20^,^21^.

Among processes based on ternary systems, the study of Thermally Induced Phase Separation (TIPS) of polymer–solvent–antisolvent in a bulk system have been widely explored to produce well interconnected porous structures, such as tissue engineering scaffolds, or different morphologies with tailored properties, such as closed or open-pore structures, spheres, powders and particles with a bead-like morphology^22^,^23^,^24^,^25^,^26^,^27^. We believe that a TIPS process, undergoing the thermodynamic laws in nanoconfined volumes, could lead to further advantages in terms of enhanced control of the phase separation phenomena and the properties of the resulting material.

Therefore, the main objective of this work is to demonstrate that, by thermally inducing the phase separation of a ternary polymer solution in a nanoconfinement, it is possible to induce an atypical yet interesting thermodynamic behavior of phase transition, leading to the formation of novel nanostructured morphologies with unexpected structural properties that could be useful in the nanomedicine field. Indeed, the concurrent presence of high-energy interfaces and conformational entropy constraints of the macromolecules under nanoconfined conditions causes profound differences in the aggregation behavior of the polymer^13^,^15^,^16^ and can influence both its morphology and its crystallinity degree. This work aims to exploit this peculiar phase transition to induce the formation of crystallized architectures, which, at the same reactant concentrations and process conditions, are not usually formed in bulk media.

Specifically, exploitation of Poly-L-Lactic Acid (PLLA) for the production of nanocapsules (NCs) of tunable size and shell thickness is carried out through a versatile and easily scalable process, based on TIPS controlled by the peculiar thermodynamics occurring in nanoconfinement. Our approach is intended to become a general strategy for the design and development of processes to produce multifunctional polymer capsules with controlled chemical composition and physical properties^28^,^29^,^30^,^31^,^32^.

In this perspective, a one-step strategy called nanoconfined-TIPS (*nc-TIPS*) is proposed to obtain a high control of the main parameters involved in TIPS, through the application of a nanoconfined droplet domain; this approach promotes the crystallization of polymer chains under unusual thermodynamic conditions, inducing, at the same time, the formation of nanocapsular structures of controlled size and shell thickness. This is accomplished by performing a quench step of a low-concentrated PLLA solvent/anti-solvent solution included in emulsions of mean droplet size <500 nm. The versatility of the proposed approach becomes evident by considering the controlled crystallinity degree, morphological properties, release mechanism and payload capability of the developed PLLA nanocapsular structures.

PLLA has been selected because it exhibits key characteristics in terms of crystal polymorphism, deriving from its chiral center in the molecular structure. In addition, being a thermoplastic, high-strength, high-modulus polymer with thermal degradation and biocompatible/bioabsorbable properties, it is a promising material for several applications. PLLA is hence one of the few commercial biopolymers of potential interest for the study of the effect of phase separation within nanoconfinements^10^,^33^,^34^,^35^,^36^,^37^,^38^,^39^,^40^,^41^.

## Results

### Observation of the spherical shape and nanocapsular structure

The production of semicrystalline PLLA nanocapsules is attained by inducing TIPS in nanometric droplets, used as a nanoconfinement.

The production process consists of the preparation through homogenization of a primary emulsion, where the disperse phase is a ternary system, polymer/solvent/antisolvent, whereas the continuous phase is made by a solution containing a certain concentration of surfactant. PLLA phase separation is induced by cooling the emulsion with water at 5 °C through a tube in tube system, which causes the rapid cooling of the emulsion at a temperature ranging from 5 to 9 °C dependence of the cooling flow rate. The detailed experimental procedure is described in the Materials and Method Section. The obtained suspension is kept at a low temperature for at least 15 minutes under continuous stirring and then centrifuged to collect the nanocapsules.

**Figure 1:**
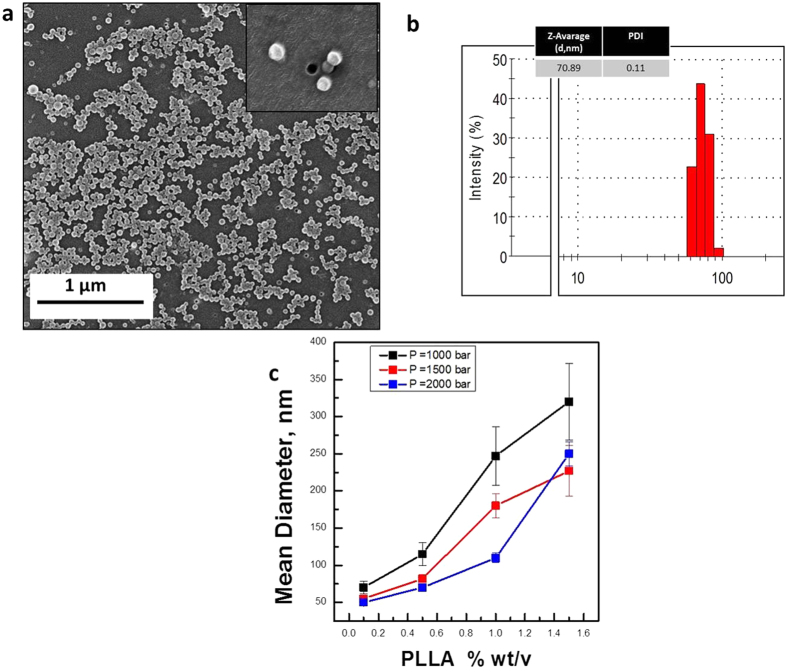
Size and Shape of PLLA Semicrystalline Nanocapsules by SEM and DLS results. (**a**) Scanning Electron Microscope images showing the morphology of nanocapsules obtained by TIPS (PLLA NCs Mean Diameter: 70 nm); (**b**) Detail of a Particle Size Distribution obtained for experiments conducted at 2000 bar and PLLA concentration of 0.5% wt/v; (**c**) The effect of polymer concentration on the mean diameter is plotted on the graph; it highlights how by reducing the polymer concentration and -consequently- Droplet Size Distribution, Mean Diameter of nanocapsules also decreases.

In Fig. 1a, Scanning Electron Microscope (SEM) images of the nanoparticles obtained from an emulsion processed at 2000 bar and a PLLA concentration of 0.1% wt/v with a mean particle diameter of 70 nm are shown (Fig. 1b). The data of mean particle size are reported in Fig. 1c as a function of the main operating conditions, homogenization pressure, polymer concentration, temperature and recirculation time.

Remarkably, the experimental results show that nanocapsular hollow semicrystalline PLLA structures are obtained only when the mean droplet diameter of the primary emulsion is below 500 nm with a polydispersity index measured by DLS of about 0.15. In contrast, when the mean droplet diameter of the primary emulsion is larger than 500 nm, non-hollow nanoparticles are obtained. In particular, polymer concentration, pressure and time of homogenization significantly influence the droplet size distribution of the primary emulsion (Fig. 1c) and therefore the formation of hollow capsules, as well as the regulation of their size and shell thickness.

Specifically, experimental results clearly show that by reducing the polymer concentration from 1.5 to 0.1% wt/v a decreasing in the mean diameter of the nanocapsules and standard deviation is obtained.

**Figure 2:**
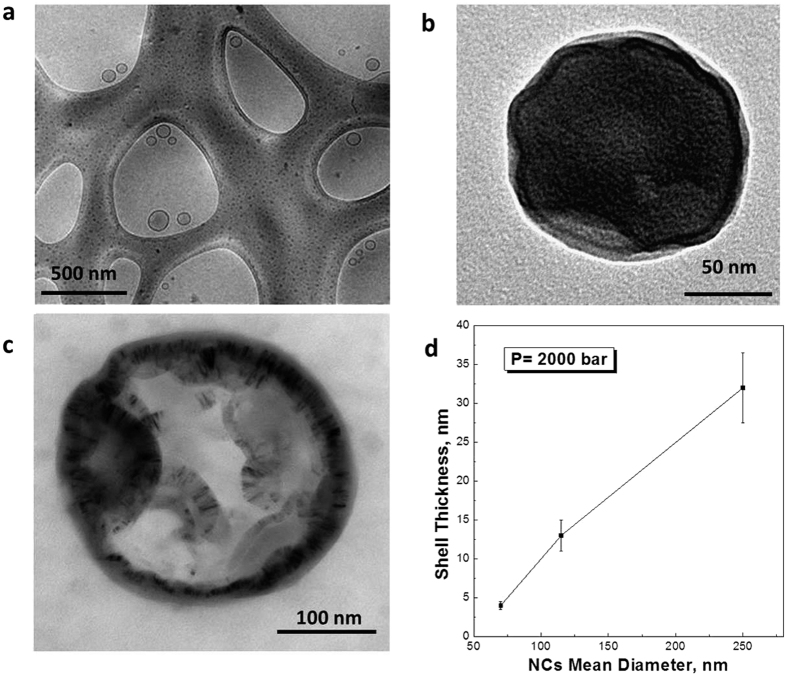
Analysis of shell thickness by TEM results. (**a–c**) Cryo –TEM and TEM nanocapsules obtained by Thermally Induced Phase Separation in a nanoconfinement by changing the polymer concentration from 0.5 to 1.5% wt/v, respectively, at a constant pressure of 2000 bar; (**d**) Graph showing the reduction of shell thickness as a function of the NCs mean diameter. A significant decreasing of mean diameter and shell thickness is observed by changing the polymer concentration.

The hollow structure of the produced nanocapsules is evident from Transmission Electron Microscopy (TEM) images, shown in Fig. 2. In particular, Fig. 2a shows the nanocapsule formation for a low PLLA concentration (1% wt/v) while in Fig. 2b an increase in capsule size due to an increase in PLLA concentration (1.5% wt/v) is reported. Figure 2c reports an enlargement of the semi-crystalline nanocapsule, highlighting the organization between crystalline phase and amorphous phase, never observed before in this type of morphologies.

Finally, Fig. 2d reports the dependence of shell thickness, evaluated from TEM images, on the mean particle size of the hollow capsules, the latter derived from DLS measurements. As it is possible to observe, the reduction in polymer concentration in the disperse phase of the primary emulsion causes a decrease in size as well as in shell thickness of the produced nanocapsules. Reported results are obtained at constant pressure and homogenization time. Related to their mean diameter, the produced semi-crystalline PLLA nanocapsules (NCs) exhibit a shell thickness of maximum 32 nm. However, by tuning the nanoconfinement and polymer concentration, their shell thickness and, consequently, loading capability can be largely modified.

Shell thickness of the nanocapsules dictates the mechanical strength and the permeability of the shell. Even if the observed shell thickness of the produced NCs is relatively small, the shell exhibits a compact structure with a low permeability value because of its semi-crystalline nature, not reported until now in capsular structures with similar thinner shell^42^. It is indirectly proved by their release properties, as discussed later in Drug loading and Release Behavior Section.

### Crystallinity grade of PLLA Nanocapsules

Despite extensive studies, crystallization behavior, crystal structure and melting behavior of PLLA are still not completely understood. Three crystalline forms (α, *β*, and *γ*) have been reported for PLLA. Crystallization from the melt or solution leads to α crystal form, which is the most common polymorph. In the α form, two chains with 10^3^ helical conformation are packed into an orthorhombic unit cell with dimensions of *a* = 10.7 Å, *b* = 6.45 Å, and *c* = 27.8 Å (along fiber axis). The *β* form is obtained under high drawing conditions and higher crystallization temperatures and it is considered to have a frustrated structure, containing three chains in a trigonal unit cell with *a* = *b* = 10.52 Å and *c* = 8.8 Å. For α and β crystal structures, melting temperatures reported are about 185 °C and 175 °C, respectively^33^. The *γ* form, recently observed, has also been observed *via* epitaxial crystallization on hexamethylbenzene substrates^33^,^43^,^44^,^45^.

As generally observed by DSC analysis, when semi-crystalline PLLA is processed, its crystallinity changes depending on the cooling rate. Typically, in TIPS processes as well as in injection molding, the crystalline phase of PLLA is only obtained at low cooling rates. In contrast, at high cooling rates, amorphous products are formed^46^.

**Figure 3:**
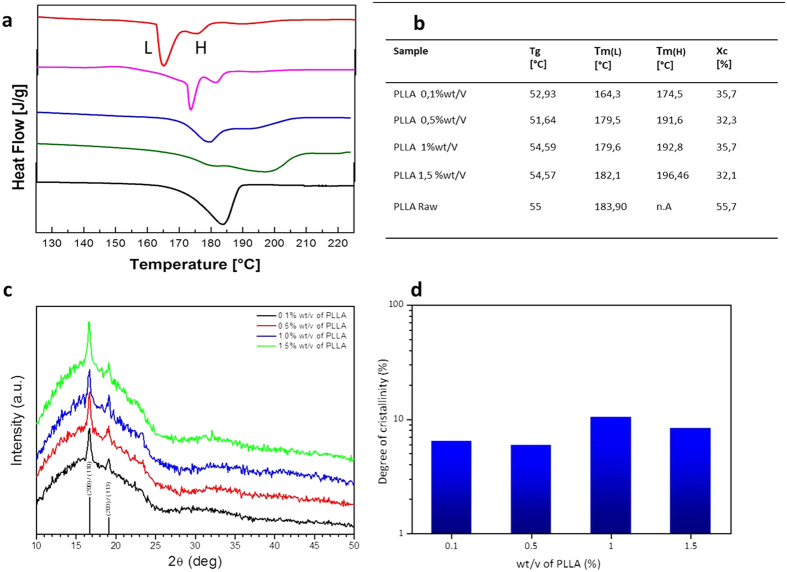
Crystallinity grade analysis by DSC and XRD. (**a**) DSC heating curves of PLLA nanocapsules at different polymer concentrations, from the top: 0.1- 0.5- 1- 1.5% wt/v–PLLA Raw; (**b**) Table reports DSC data in detail and the crystallinity grade, showing how the polymer concentration can influence crystallization phenomena (DSC heating rate: 5 °C/min); (**c**) XRD patterns at different PLLA concentrations from 0.1% wt/v to 1.5% wt/v; (**d**) Degree of crystallinity versus PLLA concentration.

In our approach, a semicrystalline morphology is obtained at very high cooling rates, more than 40 °C/min, contrary to the general observations above reported proving that a different phenomenon is interfering with the thermodynamic involved in the nanocapsule formation. In these perspectives a calorimetric study was performed and DSC curves at a cooling/heating rate of 5 °C/min obtained for the produced NCs are showed in Fig. 3a, whereas the thermal results are listed in Fig. 3b.

The average glass transition temperature (*T*_g_) of PLLA NCs ranges from about 50 °C to 55 °C and different *T*_*m*_ peaks are showed by changing experimental parameters.

Specifically, Fig. 3a shows the melting behavior of untreated raw PLLA in comparison with the PLLA NCs produced by nanoconfined TIPS.

The raw PLLA DSC curves exhibit a single and sharp melting peak at 183.9 °C, whereas dried PLLA NCs, obtained under different processing conditions, always exhibit a complex melting behavior, with multiple endothermic peaks at different temperatures.

In detail, two endothermic peaks appear in the DSC curve of NCs at 0.1% wt/v (blue curve in Fig. 3a), which can be tagged as Low and High melting temperatures (respectively, L and H in Fig. 3a). At increased polymer concentrations, the peak at low melting temperatures and its area decrease, whereas the peaks at high temperatures increase gradually up to temperatures higher than those usually observed for PLLA concentration of 1.5% wt/v.

The low-(L)- and high-(H) -temperature endothermic peaks appear when the melting rate overwhelms that of the recrystallization. A double melting peak is a common phenomenon for polymers and can lead to different interpretations. It can be due to the presence of two distinct crystals or morphological structures in the original sample, but often it is the results of annealing occurring during DSC scans, whereby less perfect crystals have time to recrystallize a few degrees above and to re-melt^38^,^47^,^48^. It so-called melt-recrystallization model suggests that the low-temperature and high-temperature peaks in the DSC melting curve are attributed to the melting of some amount of original crystals and as well as to the melting of crystals formed through the melt-recrystallization process during a heating scan. Then, the exothermic dip between the two endothermic peaks is attributed to recrystallization: the melting proceeds through the course of the melting of original crystals, and then to recrystallization and melting of recrystallized and perfected crystals. Indeed, the melt-recrystallization model suggests that small and/or imperfect crystals change successively to more stable crystals through a melt-recrystallization process leading to a competition between melting and recrystallization. In addition, it has also been reported^49^ that this crystallization behavior could also be interpreted as characteristic of a given lamellar thickness. Lamellar structures, due to the melting procedure and polymeric material, can range from hundred angstroms to several hundred angstroms and the extreme thinness of these lamellar crystals causes their melting point to be depressed below the typical melting temperature. In particular, samples that are crystallized by quenching or slow cooling from the melt to room temperature will have crystallized to a considerable extent at a high degree of undercooling from their typical melting temperature. Since crystallization at low growth temperatures produces very thin lamellae, such samples contain large fraction of low melting crystals that cause melting depression.

However, even if these observations have been reported for crystallization from a melted polymer in a macroscopic system they could be considered valid, taking into account the intrinsic differences existing by acting in a nanoconfinement.

The overall results hence suggest that the TIPS process can be dramatically modified in a nanoconfined system up to induce crystallization. Indeed, in our system, differently from conventional TIPS processes, crystallization appears to be controlled not only by cooling rate but also by the presence of the antisolvent and of the geometrical nanoconfinement due to constraint of the polymer within the primary emulsion droplets, both energetically reducing the mobility of the polymer and increasing polymer-polymer interactions in solution.

This evidence shows that by inducing TIPS of a selected ternary solution within a nanoconfinement, the formation of the polymer chain can still occur leading to a high crystallization behavior and, in some cases, to a less stable α crystal or thinner lamellae, compared to those obtained by traditional TIPS.

Further confirmation that the crystallization induced by the polymer nanoconfinement in the primary emulsion droplet can be derived when considering the behavior previously observed in twisted and curved crystals and the different proposed interpretations. Indeed, our results clearly show the curved shape of the nanocapsular shell (Fig. 2c), presuming the coexistence of curved crystals within the shell. In this case, explanations of crystallization behavior belonging to the produced nanocapsules can be again associated with the crystal growth in the confined and curved volume of a droplet, where a TIPS process, occurring at curved liquid/liquid-droplet interface, can bring to unusual polymer solution crystallization^50^,^51^,^52^. This peculiar thermal behavior can indicate, therefore, that the crystallization path is confinement dependent. We have hypothesized that might be some “defects” in the shell of the NCs due to the fast cooling. However, results obtained at 1.5% wt/v of PLLA show that even a shift of the melting Temperature at a higher value can be observed. Indeed, the higher concentration reflects a higher presence of the polymer chain within the droplets leading to an increase of the lamellae packaging similar to domains with a larger thickness. Starting by previous considerations made on different polymer systems^53^,^54^,^55^ in solution or melting, it may be asserted that such a high melting temperature can derive from the confinement of the PLLA chains, thereby requiring a higher thermal energy actually to melt them.

To explore the actual crystalline status of our PLLA nanocapsules, we analyzed them by X-ray diffraction.

Figure 3c shows the X-ray diffraction patterns obtained from PLLA at different polymer concentrations when the primary emulsion is processed at a fixed pressure of P = 2000 bar. The two main peaks at 2θ ~ 16.60° and 2θ ~ 19.00° match with the same reflections of the PLLA powder (reported in Fig. S2 of the Supplementary Information) and allow us to index them as the (200)/(110) and (203)/(113) of the optical pure PLLA α crystalline form, respectively^33^. By changing the PLLA concentration from 0.1 wt/v % to 1.5%wt/v, no changes in the position of the main peaks are observed. It is important to point out that, at a melting temperature T_m_ of 165 °C, other crystal forms could have been induced. However, XRD patterns reported in Fig. 3c only show peaks related to the α crystalline form, definitely excluding the presence of different crystalline phases. The degree of crystallinity of the PLLA nanocapsules is evaluated, as described in the Materials and Method Section, showing a slight variation in the degree of crystallinity by changing the polymer concentration (reported in Fig. 3b).

### Drug Loading and Release behavior

**Figure 4:**
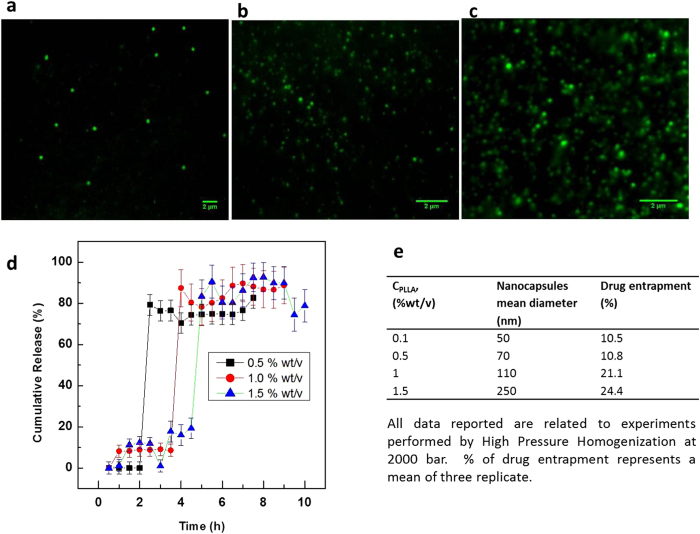
Loading capability and release mechanism. (**a–c**) STED high-resolution images of randomly dispersed fluorescence of (**a**) 250 nm, (**b**) 115 nm and (**c**) 95 nm. (**d**) Effect of polymer structure and drug release characteristics. Dye release profile of loaded nanocapsules as a function of size and shell thickness for different % of dye loadings. The graph also confirms the release mechanism typical of micro and nanocapsules.

The loading capability and release mechanism of the produced nanocapsules are reported in Fig. 4a–c, where nanocapsules having different diameter are observed by Stimulated Emission Depletion (STED). The peculiar behavior of the release mechanism is showed in Fig. 4d. The cumulative release is conducted from nanocapsules of different size and shell thickness. In particular, the cumulative dye release from SC-NCs is displayed for nanoparticles of size ranging from 70 to 250 nm.

In the cumulative curves, the real release can be seen as starting at different times, according to the shell thickness of the nanocapsules. For higher polymer concentrations, a first slight release is observed, maybe due to the absorption of the dye within the polymer shell. Curves in Fig. 4d reveal a release mechanism typical of a nanocapsular structure by degradation of the thin wall of the capsules leading to all-or-nothing cargo release. This property is crucial for delivery vehicles. Indeed, NCs could be later treated to become responsive to the local environment, in order to retain their cargo until their target is reached and complete release should take place. In this framework, it is important to point out that this burst release via wall erosion can be regulated by tuning the shell thickness of the nanocapsules through variation of polymer concentration and cooling rate.

In order to investigate the payload capability of the nanocapsules, other compounds, besides Chromeo 488 and Alexa Fluor 488, were incorporated in the developed nanocapsules, such as Rhodamine B, Nile Red and Gadolinium Chloride. The sample prepared at higher PLLA concentrations, besides showing an increase in their mean diameter, displays a slight increase in the loading capability of hydrophilic compounds to about 10%, calculated on a weight percentage basis. A different situation is observed in the case of lipophilic compounds or metal nanoparticles, where higher encapsulation efficiency is reported (more than 80%). The loading capability of the process under different condition is showed in Fig. 4e. Details about the study of the release mechanism are reported in the Materials and Method Section.

## Discussion

Generally, TIPS technique is based on changes in thermal energy to induce the de-mixing of a homogeneous polymer solution into a two- or multi-phase system domain. In a classic scenario, when the phase separation occurs, the homogenous solution separates into a polymer-rich phase and a polymer-poor phase, usually either by exposure of the solution to another immiscible solvent or by cooling the solution below a binodal solubility curve, where a liquid–liquid phase separation or solid–liquid demixing mechanism can occur. After that, the solvent is extracted by lyophilization and, depending upon the system and phase separation conditions, different morphologies and characteristics of the materials can be obtained. In this framework, PLLA has been largely studied to produce two main types of morphologies by TIPS: *globular-like* and *membrane-like.* However, PLLA is a slow-crystallizing material, and crystallization phenomena of PLLA by TIPS are not very effective regarding control of process-time and material properties.

**Figure 5:**
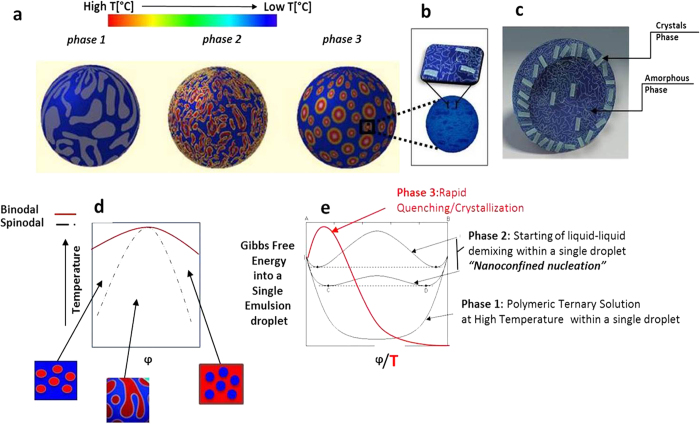
Schematic representation of thermally induced phase separation (TIPS) of a polymer solution in a nanoconfinement. (**a**) Phase morphologies influenced by a nanoconfinement. Phase separation patterns are showed by cooling the nanoconfined system at a quench temperature. In detail, a narrow DSD is obtained by High-Pressure Homogenization with a droplet mean diameter of about 500 nm and a PDI of 0.15 (phase 1). Each droplet containing a polymer solution is cooled below Cloud Point condition. At a certain temperature, phase separation occurs within the droplet and pushes them in a higher energy state similar to a spinodal phase (phase 2). Because of the nanoconfinement, polymer chains have a reduced number of available configurations to reduce their energy, so that a transition to a more orderly structure is preferred. When keeping cooling the system, a further segregation is originated such that the bending of the polymer chains forms one or more nanocapsular structures at low free energy (phase 3). (**b,c**) Schematic representation of nanocapsules and cross section highlighting crystal lamellae within the shell. (**d**) Standard polymer segregation and phase diagram displaying spinodal curves, within the coexistence curves and upper critical point. (**e**) Energy content of the different phase steps to obtain nanocapsules. Starting from a ternary solution in an emulsified system (phase 1), corresponding to a ∆G <0, no heterogeneity appears from the system before point D at a lower T, where signs of liquid-liquid miscibility gap can be detected and nanoconfined nucleation starts showing an increase in ∆G, while the presence of a nanoconfinement drives the polymer solution to a chain alignment at atypical conditions to reduce the free energy (phase 2). When keeping cooling the system, (phase 3) an interconnected system appears to form a nanocapsular structure.

The thermodynamic scenario significantly changes when a nanoconfinement is applied, as schematically depicted in Fig. 5.

We hypothesize that, when TIPS is performed in a nanoconfinement, the polymer solution experiences an entropy variation of crucial importance for the free energy change accompanying the formation of morphologies and crystal domains^16^,^56^,^57^. The constraints applied to a free unperturbed polymer chain reduce the number of available conformations of the chain and introduce additional interactions between the polymer segments and the surface.

Indeed, in the case of macromolecule-containing systems, the high molecular weight and the possibility for the long chain to experience a number of conformations are additional features that should be taken into account when studying thermodynamics or properties of confined systems. Among other properties, this reduction influences the conformational entropy of the polymer chain, determining a change in many characteristics of a polymer, such as its mobility and its miscibility.

Such information is of fundamental importance to evaluate the effect of the confining conditions compared to the traditional thermodynamic of processes such as TIPS and when the presence of the interface starts to influence the number of conformations allowed for the polymer chain in phase separation processes, i.e. when the entropic reduction becomes considerable. Indeed, by varying the degree of confinement of the system, the reduction of conformational entropy can make energetically favored chain conformations characterized by a higher order degree. Thus, the level of confinement can be used as a controlling parameter to obtain peculiar yet interesting morphologies of the phase separated systems, due to its influence on the thermodynamics of ternary systems and, consequently, on the mechanism of the phase separation process.

As reported in the literature for spherical confinement^16^,^17^, the contribution of the entropic penalty is complex to assess because of excluded volume effects. Indeed, two factors are influencing the coordination number and, hence, the conformational entropy of the polymer in the vicinity of the interface: the excluded volume of the polymer segments and the excluded volume of the obstacle.

By the above principles, here we experimentally study the effect of quenching on PLLA-Dioxane-Water ternary system^58^, not in a macroscopic environment how reported for the traditional TIPS, but confined in the droplets of a water-in-oil nanoemulsion (nanoconfined TIPS).

Briefly, as reported in Fig. 5a–c, a polymer ternary solution, at a fixed temperature and selected composition, is used as a disperse phase in an oil-continuous phase^59^,^60^. Typically, keeping the temperature constant and emulsifying the system under high shear rates, a mean droplet size lower than 500 nm is obtained. At this threshold, every emulsion droplet becomes microreactor containing a ternary polymer solution and the cooling of the system starts, causing the formation of a metastable region. Under these conditions induced in the nanoconfinement, we believe that PLLA molecular chains can overcome the chemical potential barrier and chain folding can also occur at a high cooling rate, to maintain the thermal dynamic equilibrium. In detail, if the polymer solution is trapped in a nanoconfinement, the system will be unable to adopt all its possible assembled conformations fully through thermal fluctuations, with the consequence that chain segment alignment will probably correspond to the lowest available energy status of the polymer system.

Subsequently, some folded chains immediately aggregate, leading to the formation of crystal nuclei within the droplet. However, at this point, further aggregation phenomena are prevented by a large energy barrier due to intersegment attractive and repulsive interactions, volume effects and presence of antisolvent: only the development of interconnected domains characteristic of spinodal decomposition can proceed^50^,^51^,^61^.

Our experiments are performed in the low polymer concentration region (Fig. 5d), where the formation of a solvent rich phase, as well as of globular-like morphology, is facilitated by decreasing the temperature. Finally, the preformed crystal nuclei adopt an interconnected nanocapsular structure to reduce the excess of free energy, instead of forming an extended or isolated structure. The explanation of this behavior, illustrated in Fig. 5e, lies in the reduced number of available conformations deriving from the nanoconfinement and the presence of the antisolvent.

In the framework of traditional thermodynamics, we can make some considerations: when TIPS is carried out in a macroscopic system, polymer chains can adjust to an infinite number of configurations to reduce their free energy, with the consequence that the formation of a crystalline structure is not energetically preferred at a high cooling rate^33^,^34^. In a nanoconfined system, instead, chains can adjust to a reduced number of configurations to decrease the excess of free energy, with the consequence that chain folding could become the preferred conformations able to bring the system to a reduction of the entropic penalty.

In summary, TIPS in a flexible nanoconfinement can promote a spinodal-like regime where even the smallest fluctuations can grow as crystal nuclei because of the shallow energy barriers and drive the formation of nanocapsular structures when surface energetic contributes to the formation of an interface are predominant, in comparison with the volume energetic contributes. Briefly, by increasing the energy of the polymer/solvent/antisolvent mixture at the interface by means of a spherical nanoconfinement, the system is affected by an entropic penalty. By cooling the system in this unfavorable state, it is possible to force the polymeric chain to a crystalline configuration (chain folding) and to a nanocapsular structure, shifting the system to a condition where the thermodynamic barrier for the formation of a partial crystalline phase is lower than that for the formation of a full amorphous phase. This process suggests that, within a nanoconfinement, structural changes in a polymer containing a crystallizable component compete with those occurring due to amorphous microphase separation.

Even if our hypotheses have been referred to the case of a single chain in a sphere with radius comparable to the gyration radius of the polymer in free solution, they can be consolidated and extended starting by some recent analysis by a phenomenological theory and Monte Carlo simulations, provided by Winkler *et al*.^62^,^63^,^64^, on a colloidal polymer system in a spherical confinement. In their work, it is shown that the interplay of finite size and surface effect for fluids confined inside a sphere can strongly enhance the miscibility of the mixtures, on dependence of the wall potentials at the confining surface and the tunability of the wetting properties leading to very special shape of the loops observed for the chemical potential of the colloids as a function of their packing fraction. In particular, the authors report that effective free energy as a function of the number of colloidal particles in a spherical cavity and, at a certain chemical potential of the colloids, a two-phase coexistence regime is reached in the case of complete wetting so that all colloidal particles are very close to the confining surface enabling the formation of a precursor of a wetting layer. Additionally, Winkler *et al*. emphasize that this regime corresponds to the top of the energetic barrier characterized by a core-shell structure. On the contrary, in the case of almost neutral walls we have a reduction of the interfacial area and the interface separating the polymer rich phase and the colloidal rich phase and its curvature represents the maximum of the effective free energy. This case is similar to the one hypothesized for our system as reported in Fig. 5 of this work and where probably there is no wetting between the emulsion droplet surface and the polymer-rich phase, and the smaller the polymer-rich nanodroplets, the larger curvature effects on the interface tension between the unmixed phases come into play.

Our observations demonstrate for the first time how morphologies resulting from TIPS within a nanoconfinement can be extremely different from those obtained from traditional TIPS in a macroscopic environment. These theoretical considerations are illustrated here for the case of PLLA formation, but could be generalized and applied to many other polymer systems involved in phase separation processes. Moreover, the model we propose contributes to advance the understanding of how polymorphism of some polymers could be influenced by an energetic nanoconfinement and how this knowledge could influence both the understanding of fundamental thermodynamics and technological advances in design and scale-up of processes. However, the acquisition of more information on the structural properties and fundamental transition of the macromolecules by diffraction techniques will be the step forward towards a deep knowledge on this system to allow a full theoretical interpretation of this complex phenomena.

## Conclusions

Several theoretical studies have shown that phase separation of a polymer system in a nanoconfinement significantly differs from that occurring in bulk, because of changed thermodynamic conditions.

We demonstrated that it is possible to promote the aggregation behavior of PLLA polymer chains through a thermally induced phase separation (TIPS) process in a nanoconfinement to obtain semicrystalline nanocapsules in a controlled and dynamic fashion.

By confining PLLA-dioxane- water ternary system in the droplets of a water-in-oil nanoemulsion and rapidly quenching the system, it is possible to produce these morphologies, not occurring in the bulk systems under similar process conditions.

By varying the nanoconfined domains, polymer chains can adjust to a reduced number of configurations to decrease the excess of free energy, with the consequence that chain folding become the preferred conformations to reduce the entropic penalty of the system.

The nanoconfinement strongly influences the surface properties and morphology of the nanocapsular structures and, in particular, enables to modulate shell thickness and its crystallinity degree and capsule size. It has significant implications for release mechanism and loading capability of the nanocapsular structures as shown for different hydrophilic and lipophilic payload compounds.

## Material and Method

### Ternary Solution Preparation

An homogeneous ternary solution polymer solvent/antisolvent is prepared using PLLA as a polymer (pure Poly–L–Lactic acid or Resomer^TM^L 209 S purchased by Evonik Industries), Diethylene dioxide (1,4–dioxane purchased by Sigma–Aldrich of analytical quality and without any further purification) as a solvent for the chosen polymer and Milli–Q water as an antisolvent. The constant dioxane to water weight ratio of 87/13 is chosen based on previous literature studies on the same system. The concentration of PLLA is chosen to range between 0.1–1.5% wt/vol. The components are added in an Erlenmeyer bulb, immersed in a silicone oil bath on a stirring hot plate. A thermocouple is used to control the oil bath temperature, set at 116 °C, in order to allow the polymer to dissolve in the dioxane/water mixture; the system is maintained at this temperature for at least 3 hours (depending on the polymer concentration). The system is kept under continuous stirring (300–350 rpm), to facilitate the dissolution of the solid polymer pellets in solution and to keep the system homogeneity. The temperature of 116 °C is higher than both the boiling points of dioxane (101 °C) and water (100 °C); therefore, during the dissolution of the polymer, evaporation of the low molecular weight species is expected, with a consequent undesired change in system composition. To avoid this drawback, a glass Liebig condenser is installed on the top of the Erlenmeyer bulb containing the solution allowing condensing the vapors generated by heating the system, restoring the desired solution composition. After several hours, when the solution appeared transparent, without stopping stirring, the system temperature is finally lowered at the preferred T and maintained at this value for at least one hour before starting the experiment, to stabilize the homogeneous solution obtained.

## Experimental procedure

Semicrystalline PLLA nanocapsules (NCs) are produced in a cost-effective way and a large quantity through Thermally Induced Phase Separation (TIPS) starting by a preformed emulsion. The process is performed in a continuous mode for preparing stable, dispensable, nanosized, biopolymer nanocapsules. To start, a ternary solution at a selected temperature T_1_ ranging from 30 to 70 °C is added at a certain T to a continuous oil phase containing a selected surfactant (Span80, Tween 20) at the same temperature, with a surfactant concentration ranging from 0.1 to 5% wt/v. The emulsion is treated by ultrasonication (Branson Sonifier 450 cell disruptor–25 KHz max 5 min–max 60% Amplitude) at constant temperature and directly flowed in the High-Pressure Homogenizer (Microfluidics M110P) with a Pressure ranging from 1000 to 2000 bar. After recirculating the emulsion for 10 minutes at a high shear rate, the cooling steps is performed. Emulsion is unloaded and kept under stirring at the same temperature, then directly injected in a capillary tube covered with a cooling jacket containing cold water at 5–6 °C avoiding contact between water and polymer solution. Inlet and outlet temperatures are measured by a thermocouple. The obtained suspension is kept at 7–8 °C for at least 15 minutes up to 30^59^,^60^.

### Characterization of the nanocapsules

The suspension is also centrifuged twice at 55.000–80.000 rpm for 45 min 4 °C by Ultracentrifuge Optima MAX-XP by Beckman Coulter. Hydrodynamic diameter and polydispersity index of the PLLA nanoparticles are measured using a particle size analyzer (Nano-Zeta-sizer S90, Malvern Instruments, UK). The degree of purification is evaluated by between FT-IR of PLLA powder and PLLA nanocapsules. To study the morphology, nanocapsule suspensions are usually submitted to ultrafiltration under vacuum on a polycarbonate isopore membrane (cut-off 50 nm) to remove the residual water or surfactant. The morphology of the nanoparticles is observed under a Field Emission -Scanning Electron Microscope (FE-SEM) Ultraplus Zeiss–EHT max 30 KV. To improve conductivity, SEM samples are coated with a thin layer of metal gold, chrome or platinum-palladium ranging from 3 to 7 nm.

The lyophilization of nanocapsules is performed using a freeze-dryer Christ, 1-4LSC. Briefly, a Freezing step for 3 hours at 80 °C with a cooling profile of 1 °C/min is applied, sublimation at a shelf-temperature of 6 °C and pressure of 0.85 mbar for at least 24 h and finally, secondary drying at 25 °C and 0.03 mbar for 5–6 hours. Trehalose or sucrose ranging from 1–3 wt/v% is added as cryoprotector if necessary. Dried particles are also observed by Field Emission-SEM.

The conventional transmission Electron Microscope (TEM) images are obtained at 80 and 200 kV with a FEI Tecnai 200T under low-dose conditions. Nanocapsular structures are also observed by CRYO-TEM under liquid Nitrogen and CRYO- Tomography on the same instrument. Cryo section of the instrument is purchased by GATAN Inc. For cryo-TEM experiments in the TEM, commercially available lacey carbon membranes on 300-mesh copper grids are used. A sample of the dilute nanocapsule solution is applied to the grid, blotted to a thin film with filter paper, and immediately plunged into liquid propane/ethane (−186 °C) using a vitrifrication system Vitrobot purchased by FEI. A Gatan model 626 cryo-transfer station and cryo-holder are used to transfer the grid containing the vitrified suspension into a TEM Tecnai FEI 200 kV (cryo-tomography video is reported as supplementary file).

### Calorimetric studies

Thermal properties of PLLA semicrystalline NCs are studied using differential scanning calorimetry (DSC) (TA Instruments) at a heating rate of 3, 5, 10 °C/min under nitrogen atmosphere. Glass transition temperature (Tg) is determined as the point of half Cp (heat capacity) extrapolated, while the melting temperature (Tm) is determined as the peak temperature. The degree of crystallinity (Xc) is calculated as X_c_ = ∆Hm/∆Hm^0^; where ∆Hm is an enthalpy of melting of PLLA and ΔHm^0^ is the enthalpy of melting of 100% crystalline PLLA. The melt enthalpy considered for an enantiopure PLA of 100% crystallinity (ΔH°_m_) is 93 J/g; it is the value most often referred to in the literature although higher values (up to 148 J/g) have also been reported. Calorimetry measurements are conducted on the obtained nanocapsule suspension and of the freeze-dried particles.

### X-ray diffraction studies

X-ray diffraction data are collected at room temperature from a PLLA nanocapsule suspension deposited onto Si zero-background substrates as well as from PLLA in powder. XRD measurements are performed with a D8 Discover-Bruker diffractometer equipped with a Cu source (λ_Kα1_ = 1.54056 Å and λ_Kα2_ = 1.54439 Å), a Goebel mirror, a Eurelian cradle goniometer, and a scintillator detector. XRD patterns are collected under ambient atmosphere at a fixed incident angle of 5° while moving the detector over the 10–60° range with a step size of 0.05. In this context we used X-Ray diffraction to identify the crystalline/amorphous nature of PLLA nanocapsules and the extent of crystallinity present in the samples.

The ratio between the integrated intensity of the diffraction peaks (crystalline contribution) and the whole area under the entire XRD pattern (crystalline and amorphous contribution) is evaluated to determine the crystallinity degree. No additional background is present due to the zero contribution of the Si substrate^65^. The results are reported in Fig. S3 and Fig. 3d.

### *In vitro* release

The release studies were performed by ultrafiltration techniques. The separation of free drug from incorporated drug was obtained by ultracentrifugation at 80.000 rpm and 4 °C. The suspension (5–7 ml) was added directly into a stirred ultrafiltration cell (Amicon Stirred Cell setup, including selector valve and reservoir accessories, purchased by Millipore.) containing PBS (pH 7.4, 37 °C) and moderately stirred. At specified time intervals aliquots of the release medium (1–3 ml) were filtered through the ultrafiltration membrane with a molecular weight cut-off point of 150 000 Da. The withdrawn sample was replaced with equal volumes of fresh dissolution medium. Specific dye at λ max (Spectrofluorometer, Horiba) was measured. The percentage drug released at each time point was corrected for dilution by sample replace. Experiments were repeated at least 3 times. The results are reported in Fig. 4d.

## Additional Information

**How to cite this article**: Torino, E. *et al*. Synthesis of semicrystalline nanocapsular structures obtained by Thermally Induced Phase Separation in nanoconfinement. *Sci. Rep.* **6**, 32727; doi: 10.1038/srep32727 (2016).

## Supplementary Material

Supplementary Information

Supplementary Video
