# Supplementary material for: Synthesis of semicrystalline nanocapsular structures obtained by Thermally Induced Phase Separation in nanoconfinement
Source: Sci Rep. 2016 Sep 8;6:32727. doi: 10.1038/srep32727 (PMC5015022; doi:10.1038/srep32727)
Supplement: Supplementary Information [file srep32727-s1.pdf]

SUPPLEMENTARY INFORMATION

Synthesis of semicrystalline nanocapsular structures obtained by Thermally Induced Phase Separation in nanoconfinement

Enza Torino[a,b], *Rosaria Aruta*[a,c], *Teresa Sibillano*[d], *Cinzia Giannini*[d], *Paolo A. Netti*[a,b,c]*

[a] Istituto Italiano di Tecnologia, Center for Advanced Biomaterials for Health Care IIT@CRIB, Largo Barsanti e Matteucci 53, 80125, Naples, Italy.

[b] University of Naples Federico II, Interdisciplinary Research Center of Biomaterials, CRIB P.le Tecchio 80, 80125, Naples, Italy.

[c] University of Naples Federico II, Department of Chemical, Materials and Industrial Production Engineering, P.le Tecchio 80, 80125, Naples, Italy.

[d]CNR - IC Istituto di Cristallografia, via Amendola 122/O, 70126 Bari - Italia

corresponding email: paolo.netti@iit.it

Pressure, bar	C, %wt/V	Mean Diameter, nm	SD (±)	PDI
2000	0.1	50	2.375	0.095
2000	0.5	70	3.85	0.11
2000	1	110	6.6	0.12
2000	1.5	250	16.25	0.13
1500	0.1	55	2.6125	0.095
1500	0.5	82	4.92	0.12
1500	1	180	16.2	0.18
1500	1.5	227	34.05	0.3
1000	0.1	70	8.75	0.25
1000	0.5	115	15.525	0.27
1000	1	247	39.52	0.32
1000	1.5	320	51.2	0.32

Table S 1: Table reporting details about the conducted experimental campaign and the related mean diameter. Last columns within the table show Standard Deviation (SD) and Polydispersity Index (PDI) as obtained by Dynamic Light Scattering results. All the experiments have been performed under same conditions of temperature and cooling rate

Intrinsic viscosity and Molecular Weight of PLLA Nanocapsules.

Because PLLA for the preparation of the nanocapsules is dissolved for at least 3 hours in a dioxane / water mixture (containing 13 % water) at temperatures well above the boiling temperature of water, the intrinsic viscosity of the polymer was compared with the PLLA after the dissolution and the production of nanoparticles. For the linear and unbranched polymers such as our PLLA, the viscosity of a diluted polymer solution is directly correlated to the viscosity average of the molar mass M_{vis} by the Mark-Houwink equation (MHE).

$$[\eta] = K \cdot M_{vis}^{\alpha}$$

Viscosity of the samples were measured in chloroform and THF for PLLA using Ubbelohde viscometer in a thermostated water bath at 30±0.1°C. Intrinsic viscosities of the samples were obtained by the extrapolation of the plots of the inherent and reduced viscosities against concentration of samples,

$$M_{vis} \approx M_w$$

The concentration-dependent viscosities were extrapolated to zero concentration to deduce intrinsic viscosity. Because reliable MHE parameters K and α can be found in literature for PLLA, the molar mass average can be calculated out of the inherent viscosity measurements. One pair of Mark-Houwink parameters K and α is only valid for a certain solvent at a defined temperature. Mark-Houwink parameters used in this work are given below[1]:

M_{vis}: K = 5.45 × 10^{-4} dL/g and α = 0.73 in chloroform at 30 °C

The Intrinsic Viscosity measured was about 2.9 dL/g for both the raw samples and the treated ones.

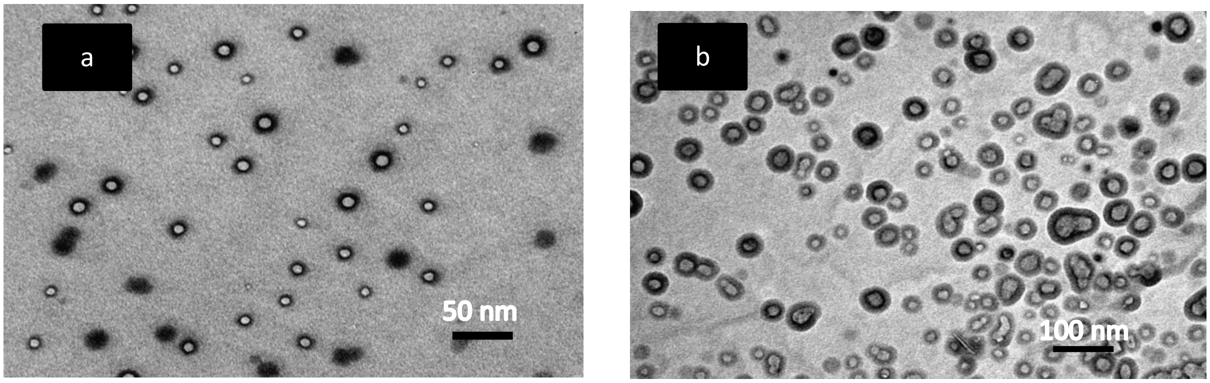

Figure S 1: TEM image of nanocapsules obtained at (**a**) 0.1 and (**b**) 0.5 %wt/v PLLA. Particle Size Distribution: **a,** 20 nm and **b,** 70 nm

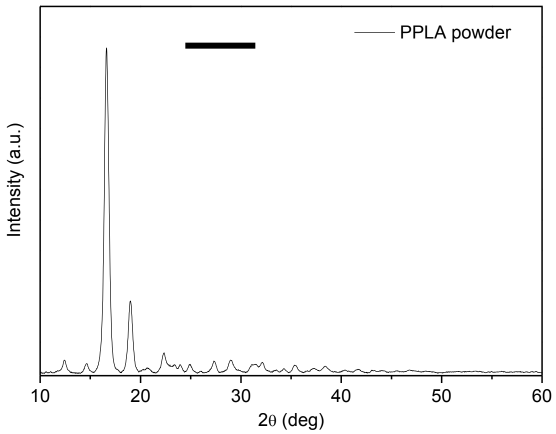

Fig. S2: Pattern of PLLA powder.

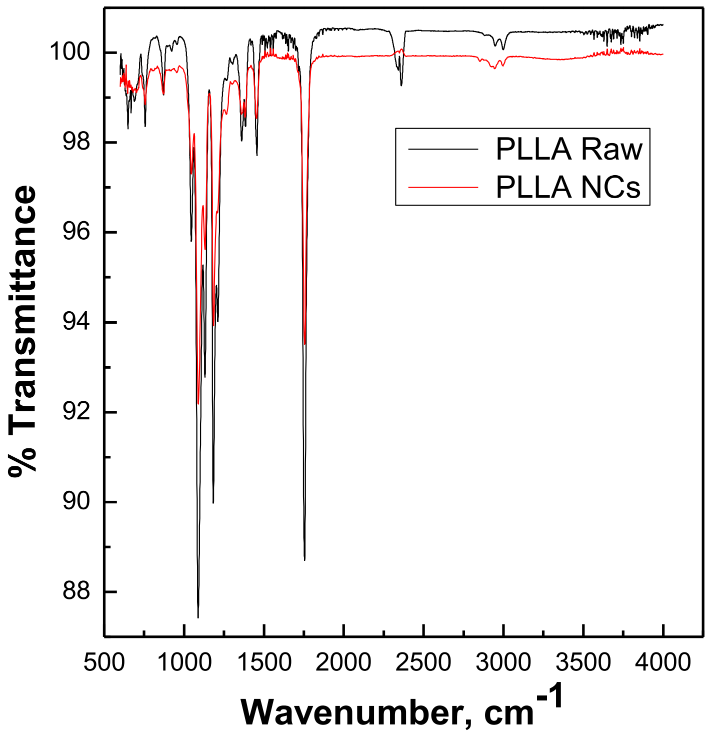

Figure S3: Comparison between FT-IR PLLA powder and PLLA nanocapsules. Observing the enlargement of the figure an additional peak related to the crystalline structures appears.

1 Lee, J. S., Kim, S. C. & Lee, H. K. Intrinsic viscosity and unperturbed dimension of poly(dl-lactic acid) solution. *Macromolecular Research* **16**, 631-636, doi:10.1007/BF03218572 (2008).

VIDEO LEGEND

3Dstructure of PLLA Nanocapsule obtained by nanoconfined TIPS (nc-TIPS) reconstructed by Cryo-Transmission Electron Tomography
